# Comparison of the Surgical Results of Ventral and Dorsal Dartos Flaps in Tubularized Incised Plate Urethroplasty for Distal Hypospadias: A Randomized Clinical Trial

**DOI:** 10.5152/tud.2025.25012

**Published:** 2025-06-24

**Authors:** Fateme Tahmasbi, Javad Aliasgarzadeh, Mohsen Mohammad-Rahimi, Behzad Lotfi

**Affiliations:** 1 Research Center for Evidence-Based Medicine, Iranian EBM Centre: A JBI Centre of Excellence, Tabriz University of Medical Sciences Faculty of Medicine, Tabriz, Iran; 2 Social Determinants of Health Research Center, Health Management and Safety Promotion Research Institute, Tabriz University of Medical Sciences Faculty of Medicine, Tabriz, Iran; 3 Pediatric Health Research Center, Tabriz University of Medical Sciences Faculty of Medicine, Tabriz, Iran; 4 Department of Urology, Tabriz University of Medical Sciences Faculty of Medicine, Tabriz, Iran

**Keywords:** Congenital abnormalities, hypospadias, surgical flaps, urogenital abnormalities

## Abstract

**Objective:**

Hypospadias is one of the most common congenital anomalies of the urogenital system. Various surgical methods exist to repair this condition, all aiming to create a straight penis, ensure proper placement of the meatus, and achieve a sufficient diameter for the urethra with optimal flow. Despite following precise surgical techniques, many patients in reported studies still experience complications. The aim was to evaluate and compare the surgical outcomes of distal hypospadias repair utilizing ventral versus dorsal Dartos flaps.

**Methods:**

In this randomized clinical trial, patients who were candidates for surgical treatment were randomly assigned to 2 groups. Both groups underwent hypospadias repair using the tubularized incised plate (TIP) method. The first group received a ventral Dartos flap, while the second group received a dorsal Dartos flap for urethral reconstruction. Patients were monitored for complications, and the Hypospadias Objective Scoring Evaluation (HOSE) questionnaire was used to assess the surgical outcomes.

**Results:**

The dorsal flap group had higher complication rates, including 3 fistulas (10%), 6 cases of mild necrosis (20%), 2 cases of severe necrosis (6.7%), and 4 instances of penile torsion (13.3%). The ventral flap group had 2 cases (6.7%) of postoperative bleeding and 2 fistulas (6.7%). At the 6-month follow-up, no significant difference was observed between the 2 groups in terms of HOSE score. The need for reoperation was higher in the dorsal flap group.

**Conclusion:**

The findings suggest that the ventral Dartos flap provides better results and fewer complications than the dorsal Dartos flap in the repair of hypospadias.

## Introduction

Hypospadias is a prevalent congenital condition, affecting around 1 in 200 male newborns. It is characterized by an abnormal urethral opening on the ventral side of the penis.^1^^,^^2^ Distal hypospadias, where the urethral opening is located on the glans or proximal to it, is the most common type.^3^ This deformity not only causes functional problems, such as difficulty urinating in a straight path, abnormal appearance of the penis, and reduced sexual function and fertility, but it also leads to psychological challenges for both the patient and their parents.

The development of hypospadias may be influenced by interactions between genetic factors and environmental exposures, such as placental insufficiency and endocrine-disrupting chemicals like vinclozolin and di-n-butyl phthalate. These factors can act on polymorphic genes or induce epigenetic changes, potentially contributing to the condition’s development.^4^^-^^6^

The goal of hypospadias treatment is to reconstruct the urethra as close to its natural location as possible while correcting any curvature in the penis. An important goal in treatment is maintaining the conical shape of the glans and ensuring adequate skin coverage of the penis. This enables proper urine flow and supports natural sexual function, ultimately contributing to the patient’s self-confidence.

Various techniques have been introduced to manage distal hypospadias, but none have been definitively proven as the ideal approach, with outcomes often dependent on the surgeon’s preference. Over the last few years, tubularized incised plate urethroplasty (TIPU) has gained more acceptance among pediatric surgeons.^7^^,^^8^ This technique enables urethral reconstruction using natural urethral plates, offering a relatively straightforward procedure. It results in a postoperative urethral opening with a vertical slit-like appearance, closely resembling the normal anatomy seen in children.

In general, fistula, adhesion, stricture, unfavorable cosmetic condition, non-repair, diverticulum, and regression of the new duct are major complications of surgical repair. The result of reconstructive surgery depends on various factors such as the severity of the deformity, androgen therapy, surgical technique and time, tools, material and suture technique, urinary drainage, and antibiotics before and after surgery. The complications of hypospadias surgery are higher compared to other reconstructive surgeries.

There are still debates about which flap is the most suitable covering to reduce the complications of hypospadias surgery. This study evaluates different flap techniques to identify which yields superior results in terms of complications, function, and aesthetics. This randomized clinical trial will provide robust evidence to guide surgical practice, ultimately improving patient care and outcomes. Hence, a clinical trial was conducted to compare the surgical outcomes and the complications of distal hypospadias surgery using ventral and dorsal Datros flaps.

## Material and Methods

In accordance with the Consolidated Standards of Reporting Trials (CONSORT) 2010 guidelines, randomized clinical trial was conducted. Written consent was obtained from the patients’ legal guardians after a thorough explanation of the study’s purpose, procedures, risks, and potential benefits. Patient information was anonymized, and data were stored securely using coded identifiers. The protocol of the study was registered and approved by the Iranian Clinical Trials (IRCT) Registration Center (IRCTID: IRCT20220218054055N1). This study has been approved by the Ethical Committee of Tabriz University of Medical Sciences (IR.TBZMED.REC.1401.500 Date: 2022-10-29))

### Eligibility Criteria for Participants

Patients were eligible for the study if they had a clinical diagnosis of distal hypospadias, were at least 6 months old, and provided informed consent. Patients were excluded if they had glandular hypospadias, a coagulation disorder, recurrent immunodeficiency, prior circumcision, proximal hypospadias, chordee > 30°, diabetes, or a history of hypospadias surgery. Additionally, patients with proximal hypospadias, chordee > 30°, diabetes, or a previous history of hypospadias surgery were also ineligible for enrollment.

### Settings and Locations

This study was conducted in the Tabriz Children’s Hospital from July 2021 to June 2022. Patients were randomly divided into two groups based on even or odd numbers which was given to them in order of the visiting.

### Interventions

All patients underwent a complete preoperative examination. Glans size, penile length, and hypospadias severity were recorded using the Glans-Urethral Meatus-Shaft (GMS) score. This scale includes 3 components: the glans score (G), the meatus score (M), and the shaft score (S), referring to the visible cylindrical portion of the penis. The combined value of these scores constitutes the total GMS score. Each component is assigned a numerical value from 1 to 4, with higher scores indicating more unfavorable characteristics. A total GMS score of 12 indicates severe hypospadias, while a score of 3 or less reflects mild hypospadias.

Both groups underwent hypospadias repair utilizing the tubularized incised plate (TIP) method. The procedure was performed under general anesthesia with 2.5× magnification. Following degloving and complete chordee correction, the urethral plate was tubularized over an 8F silastic catheter using continuous 6-0 polydioxanone sutures. In group 1, a ventral Dartos flap was used as a second layer to cover the neourethral suture line. In group 2, a laterally rotated dorsal Dartos flap was applied for the same purpose. Penile shaft coverage was achieved using a ventrally rotated Byars’ flap. Glansplasty was carried out with the use of polydioxanone sutures.

Patients were discharged 48-72 hours postoperatively following dressing removal, with the catheter in place. The catheter was removed after 1 week. Follow-up assessments were conducted at 1, 3, and 6 months post surgery.

### Outcomes

Postoperatively, patients were evaluated for complications including bleeding, hematoma, surgical site infection, and urethral fistula formation. At the 6-month follow-up, surgical outcomes were assessed using the Hypospadias Objective Scoring Evaluation (HOSE) questionnaire.^9^ The HOSE is a validated scoring system comprising 5 domains: the location of the meatus, the shape of the meatus (vertical or circular), urinary stream (single or multiple), penile curvature during erection (straight or curved), and presence or absence of a urethral fistula. The maximum HOSE score is 16, and the minimum is 5. This questionnaire has been validated and shown to be reliable in Iranian clinical settings.^10^

### Statistical Methods

Descriptive statistics were used to summarize the data, including means, SDs, and frequency percentages. To compare qualitative variables between the 2 study groups, the Chi-square test—specifically the Mantel-Haenszel test—was applied. All analyses were conducted using SPSS statistical software, version 20 (IBM SPSS Corp.; Armonk, NY, USA). A *P*-value of less than .05 was considered statistically significant.

## Results

**Figure 1.**
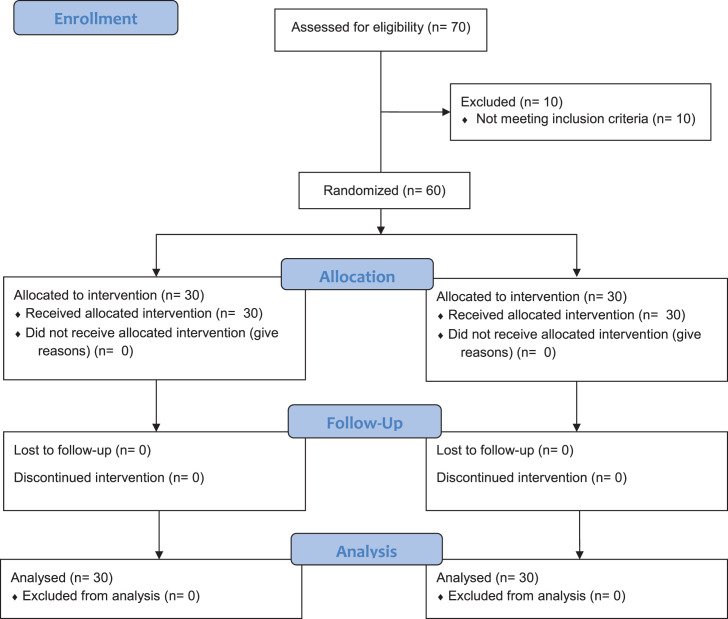
The CONSORT 2010 Flow Diagram.

A total of 70 participants were assessed for eligibility, of whom 10 were excluded due to not meeting the inclusion criteria. The remaining 60 patients were randomly assigned equally to the 2 intervention groups: 30 to the dorsal Dartos flap group and 30 to the ventral Dartos flap group. All enrolled patients completed the trial and were included in the final analysis. The CONSORT 2010 Flow Diagram illustrates the flow and progression of participants through the trial phases (Figure 1).

The mean age of patients in the ventral flap group was 21 months, compared to 29.5 months in the dorsal flap group. A comparison of GMS scores before surgery is presented in Table 1. The average total GMS score was 4.83 in the ventral flap group and 4.6 in the dorsal flap group (*P* = .5). In 3 cases originally assigned to the ventral flap group, adequate ventral flaps could not be prepared. Therefore, dorsal flaps were used in these patients, and they were reassigned to the dorsal flap group for analysis.

**Figure 2.**
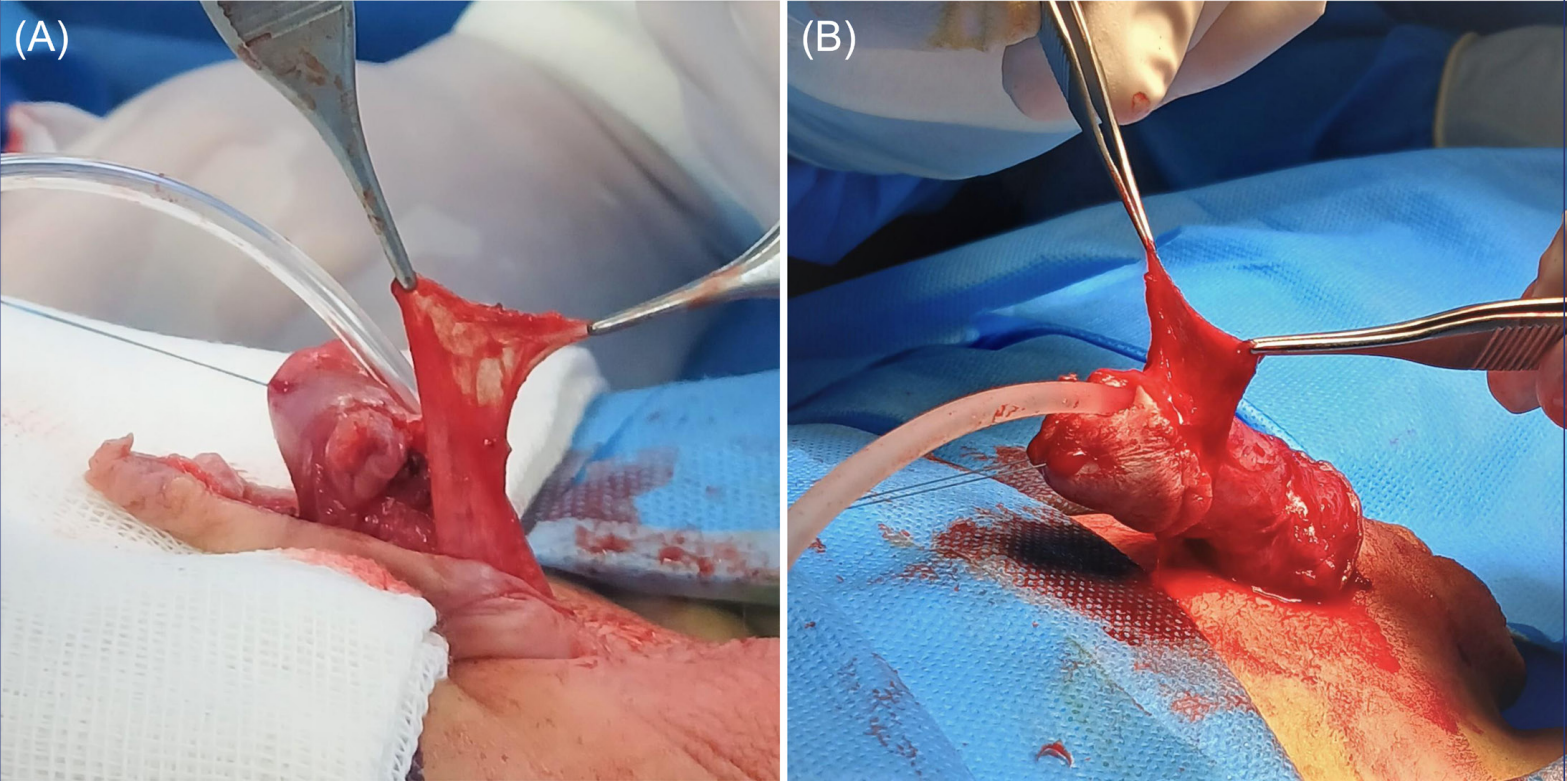
Vascularized Dartos flaps were used for urethroplasty coverage. A) Dorsal Dartos flap B) Ventral Dartos flap.

Patients were evaluated on postoperative days and again approximately 1 week after surgery for early complications such as bleeding, hematoma, severe edema, infection, and wound dehiscence. Table 2 provides a comparison of postoperative complications between the 2 groups. Severe and mild skin necrosis occurred in 2 patients (6.7%) and 6 patients (20%) in the dorsal flap group, respectively, while no cases were reported in the ventral flap group (*P* = .003). Penile torsion was significantly more common in the dorsal flap group compared to the ventral flap group (4 vs. 0 patients, *P* = .04). Two patients (6.7%) in the ventral flap group and 3 patients (10%) in the dorsal flap group developed fistulas at the 6-month follow-up visit, with a *P*-value of .643, indicating no significant difference. Lastly, secondary surgery was performed in 3 patients (10%) in the ventral flap group and 4 patients (13.3%) in the dorsal flap group (*P* = .305), again showing no statistically significant difference. Figure 2 shows intraoperative images of the dorsal Dartos flap and ventral Dartos flap used for urethroplasty coverage.

In Table 3, the outcomes of hypospadias surgery between the 2 groups are compared using the HOSE questionnaire. Regarding fistula formation, 90% of patients in the ventral flap group did not develop a fistula, compared to 93.3% in the dorsal flap group. Minor cases of single, subcoronal, or distal fistulas were observed in both groups. Additionally, the total HOSE score was slightly higher in the ventral flap group (15.50) compared to the dorsal flap group (15.13) (*P* = .12), indicating no significant difference between the outcomes of the 2 surgical techniques.

## Discussion

The choice of the optimal surgical technique for repairing hypospadias remains controversial in pediatric surgery. TIPU has gained popularity for distal and mid-penile hypospadias due to its versatility and reliable outcomes. In this study, the surgical outcomes and complication rates of ventral and dorsal Dartos flaps in TIPU were comapred for treating distal hypospadias. The findings showed no significant differences in dehiscence or fistula rates; however, skin necrosis was significantly lower in the ventral flap group (0%) compared to the dorsal group (26.7%). Penile torsion was absent in the ventral group, while 13.3% of patients in the dorsal group experienced it. Both groups reported similar outcomes regarding meatus location and shape, urinary flow, and chordee, with slightly higher HOSE scores in the ventral group; however, this difference was not significant.

The significantly lower incidence of skin necrosis in the ventral flap group can be attributed to the superior vascularity of the ventral surface of the penis, which benefits from a rich blood supply provided by both the superficial and deep penile vascular systems, as well as branches from the internal pudendal artery.^11^^,^^12^ Additionally, the Dartos fascia in this region contains a well-vascularized network. This robust vascular supply is crucial for tissue healing and viability, particularly in surgical procedures like hypospadias repair. In contrast, while the dorsal surface is still vascularized, it may not receive the same level of blood supply, making it more prone to ischemic complications when significant dissection is performed. Therefore, the ventral surface is often preferred in surgical approaches due to its superior vascularity.

In addition, the positioning and alignment of the flap can significantly affect the surgical outcome. Based on these findings, the complete absence of penile torsion in the ventral group contrasted with a 13.3% occurrence in the dorsal group. After separating the dorsal Dartos from its skin, it was pulled forward from 1 side of the penis. Although the procedure was tension-free, flap tension, particularly after shrinkage, appears to be the cause of the penile torsion in the dorsal flap group. Similarly, Al Maily et al^13^ conducted a study (N = 85) comparing the outcomes of dorsal versus ventral Dartos flaps in TIPU for repairing distal hypospadias and found 2 cases (4.8%) of iatrogenic penile torsion in the dorsal flap group. However, the type of study and randomization protocol (if any) were not mentioned.

Although current literature contains limited publications comparing ventral and dorsal Dartos flaps in TIPU, several studies are worth highlighting. First, the study by Yiğit et al^14^ retrospectively investigated the results of ventral and dorsal Dartos flaps in TIPU procedures across 2 centers. According to their findings, postoperative surgical complications occurred in 15 (33.3%) and 4 (9.1%) patients in the ventral and dorsal groups, respectively. The complications observed in the ventral group included urethrocutaneous fistula in 10 patients (22.2%) and meatal stenosis in 5 patients (11.1%). In the dorsal group, fistula occurred in 2 patients (4.6%) and stenosis in 2 patients (4.6%). Urethrocutaneous fistulas were statistically more common in the ventral-based Dartos flap group (*P* < .05). In contrast to this study, they reported that the dorsal preputial Dartos flap was more effective than the ventral-based flap in preventing fistula formation.

Ghanem and Nijma^15^ evaluated the outcomes of TIPU using a dorsal Dartos subcutaneous flap in the treatment of 49 patients with primary proximal hypospadias. Fistulae occurred in 4 cases, meatal stenosis in 1 case, and glandular dehiscence in 1 case, resulting in an overall complication rate of 12%. There were no reports of residual chordee, neourethral stricture, or urethral diverticulum.

Another study by Jia et al^16^ retrospectively compared the outcomes of using a modified meatus-based ventral Dartos flap (MBDF) with a single dorsal Dartos flap in TIPU, analyzing the complication rates based on the experiences of 2 surgeons with 356 patients. Skin necrosis and penile rotation occurred in 2.7% and 3.8% of patients in the dorsal Dartos flap group, respectively. In line with these findings, they reported no instances of ventral skin necrosis or penile rotation in the MBDF group, which was significantly less frequent than in the dorsal Dartos flap group.

Lastly, Cimador et al^17^ randomly divided 130 patients into 2 main groups, further subdividing them into 5 subgroups. Patients who received a dorsal preputial flap were stratified into 3 subgroups: a laterally twisted preputial flap, a ventrally twisted flap in a buttonhole fashion, and a preputial flap divided into 2 wings and laterally twisted. Those who received a ventral Dartos flap were divided into 2 subgroups: single-layer flap and double-layer flap. The study reported 3 cases (15.7%) of penile iatrogenic rotation occurring in the group that underwent Retik’s lateral transposition of dorsal Dartos flaps.

Since Retik et al^18^ first introduced the use of preputial Dartos for covering the neourethra, the de-epithelialized preputial Dartos flap has become a widely adopted technique. However, the lateral transposition approach has certain limitations, especially in cases of asymmetric or insufficient flap formation. In such situations, extensive mobilization of the flap may be necessary, which can potentially compromise its blood supply. Furthermore, incomplete or difficult mobilization of the flap can result in iatrogenic penile rotation. To address these challenges, variations of dorsal Dartos flap transposition, such as the buttonhole technique,^19^ double dorsal Dartos flaps^20^ and ventral Dartos flaps^21^ have been introduced.

While this study provides valuable insights into the comparative outcomes of ventral and dorsal Dartos flaps in TIPU for distal hypospadias, several limitations must be acknowledged. First and foremost, the study involved a relatively small sample size of 70 participants, which was an inherent limitation due to the rarity of the condition. This impacts the statistical power and generalizability of the findings. Second, the trial was conducted at a single center, which may limit the applicability of the results to other settings or populations. Additionally, the short follow-up duration may not capture late-onset complications or long-term functional outcomes, which are crucial for fully assessing the efficacy of these surgical techniques. Recognizing these limitations is important for contextualizing the findings and directing future research in this field.

In conclusion, no significant differences were observed in the outcomes of hypospadias surgery or the rates of fistula formation between the dorsal and ventral Dartos flaps. These findings suggest that both surgical approaches are effective for distal hypospadias treatment. However, the ventral Dartos flap may offer specific advantages in terms of reduced skin necrosis and penile torsion, making it a potentially preferable option for certain patient populations. The ventral flap is easy to harvest and has a well-established vascular supply for hypospadias repair, and it was successfully prepared in 90% of the patients. In cases with insufficient or inadequate ventral Dartos tissue, techniques such as the buttonhole or double dorsal Dartos flap can be useful alternatives to prevent complications like skin necrosis and penile rotation. Surgeons may begin the procedure using a ventral flap and make decisions about the appropriate Dartos flap based on intraoperative findings.

## Figures and Tables

**Table 1.** The Preoperational GMS Score

		Ventral Flap (N = 30)	Dorsal Flap (N = 30)	*P*
Glans size	Good	18 (60%)	19 (63.3%)	.05
	Appropriate	9 (30%)	8 (26.7%)	
	Small	3 (10%)	3 (10%)	
Meatus	Glanular	12 (40%)	11 (36.6%)	.856
	Coronal sulcus	13 (43.3%)	15 (50%)	
	Mid or distal shaft	5 (16.7%)	4 (13.3%)	
Shaft curvature	No curvature	15 (50%)	19 (63.3%)	.293
	Mild curvature (<30˚)	15 (50%)	11 (36.7%)	
	Moderate curvature (30−60˚)	0	0	
Total GMS		4.83	4.6	.508

**Table 2.** The Frequency and Comparison of the Postsurgical Complications

Complication			Ventral Flap Group (N = 30)	Dorsal Flap Group (N = 30)	*P*
Early complications	Bleeding		2 (6.7%)	0	.062
	Hematoma		0	0	–
	Infection		0	0	–
	Skin necrosis	None	30 (100%)	22 (73.3%)	.003*
		Mild	0	6 (20%)	
		Severe	0	2 (6.7%)	
	Glans dehiscence		1 (3.3%)	1 (3.3%)	
Late complications	Penile torsion	None	30 (100%)	26 (86.7%)	.040*
		<30°	0	4 (13.3%)	
		30-90°	0	0	
	Fistula		2 (6.7%)	3 (10%)	.643
	Need for secondary surgery		3 (10%)	4 (13.3%)	.305

**Table 3.** Comparing the Outcome of Hypospadias Surgery Based on the HOSE Score

Outcome		Ventral Flap (N = 30)	Dorsal Flap (N = 30)	*P*
Location of meatus	Distal glans	15 (50%)	19 (63.3%)	.255
	Proximal glans	14 (36.7%)	9 (30%)	
	Coronal	4 (13.3%)	2 (6.7%)	
	Shaft	0	0	
Shape of the meatus	Vertical	30 (100%)	29 (96.7)	.321
	Circular	0	1 (3.3%)	
Urine flow	Single	30 (100%)	29 (96.7%)	.321
	Multiple	0	1 (3.3%)	
Curvature	None	30 (100%)	28 (93.3%)	.155
	Mild (<10°)	0	2 (6.7%)	
	Moderate (10 -45°)	0	0	
	Severe (> 45°)	0	0	
Fistula	None	27 (90%)	28 (93.3%)	.393
	Single, subcoronal, or distal	3 (10%)	2 (6.7%)	
	Single, proximal	0		
	Multiple or complicated	0		
Mean total HOSE score (±SD)		15.50 (0.68)	15.13 (1.07)	.12

## Data Availability

The data that support the findings of this study are available on request from the corresponding author.
